# Carbon Quantum Dots-Doped Ni_3_Se_4_/Co_9_Se_8_/Fe_3_O_4_ Multilayer Nanosheets Prepared Using the One-Step Solvothermal Method to Boost Electrocatalytic Oxygen Evolution

**DOI:** 10.3390/ma16145115

**Published:** 2023-07-20

**Authors:** Yao Zhang, Runze Wang, Longqi Zhu, Xu Li, Caixia Sun, Haizhen Liu, Lei Zhu, Kuikui Wang

**Affiliations:** 1 Institute of Materials for Energy and Environment, Laboratory of New Fiber Materials and Modern Textile, Growing Basis for State Key Laboratory, College of Materials Science and Engineering, Qingdao University, Qingdao 266071, China; 2 Key Laboratory of New Metallic Functional Materials and Advanced Surface Engineering in Universities of Shandong, School of Mechanical and Electronic Engineering, Qingdao Binhai University, Qingdao 266555, China; 3 Material Corrosion and Protection Key Laboratory of Sichuan Province, Zigong 643000, China; 4 MOE Key Laboratory of New Processing Technology for Non-Ferrous Metals and Materials, Guangxi Key Laboratory of Processing for Non-Ferrous Metals and Featured Materials, Guangxi University, Nanning 530004, China; 5 College of Basic Medical, Qingdao Binhai University, Qingdao 266555, China

**Keywords:** oxygen evolution reaction, transition metal selenides, multilayer nanosheets, carbon quantum dots, heterostructures

## Abstract

Oxygen evolution reaction is a momentous part of electrochemical energy storage and conversion devices such as rechargeable metal–air batteries. It is particularly urgent to develop low-cost and efficient electrocatalysts for oxygen evolution reactions. As a potential substitute for noble metal electrocatalysts, transition metal selenides still prove challenging in improving the activity of oxygen evolution reaction and research into reaction intermediates. In this study, a simple one-step solvothermal method was used to prepare a polymetallic compound carbon matrix composite (Co_9_Se_8_/Ni_3_Se_4_/Fe_3_O_4_@C) with a multilayered nanosheets structure. It exhibited good OER activity in an alkaline electrolyte solution, with an overpotential of 268 mV at 10 mA/cm^2^. In addition, this catalyst also showed excellent performance in the 24 h stability test. The composite presents a multi-layer sheet structure, which effectively improves the contact between the active site and the electrolyte. The selenide formed by Ni and Co has a synergistic effect, and Fe_3_O_4_ and Co_9_Se_8_ form a heterojunction structure which can effectively improve the reaction activity by initiating the electronic coupling effect through the interface modification. In addition, carbon quantum dots have rich heteroatoms and electron transferability, which improves the electrochemical properties of the composites. This work provides a new strategy for the preparation of highly efficient OER electrocatalysts utilizing the multi-metal synergistic effect.

## 1. Introduction

Electrochemical energy storage and conversion devices are considered to be a sustainable development technology of the future [1,2]. Oxygen evolution reaction plays a key role in the application of energy storage and conversion devices, such as industrial water electrolyzers and rechargeable metal–air batteries [3,4]. Noble metal catalysts such as Iridium/Ruthenium oxide have the best overall catalytic performance in OER, but their limited availability and exorbitant price limit their extensive development and application [5,6,7]. Therefore, developing non-noble metal electrocatalysts to improve the catalytic activity of oxygen evolution reactions is an urgent assignment. Transition metal-based electrocatalysts have good competitive activity and toxicity resistance in an alkaline medium. Because of their excellent stability and abundant reserves, it has become a research hotspot to replace Ruthenium/Iridium-based electrocatalysts. Numerous studies have shown that the use of polymetallic-based electrocatalysts can achieve high efficiency by optimizing adsorption energy, increasing the stability and the electrical conductivity of the electrocatalyst [8,9]. In addition, carbon quantum dots (CQDs), as zero-dimensional carbon nanomaterials, have great potential in electrochemistry due to their fast electron transfer ability and rich specific surface area, and their abundance of heteroatoms (O, N, S) can provide ideal active sites for electrochemical behavior [10]. 

Transition metal hydroxide [11,12], oxide [13,14], sulfide [15,16], selenide [17,18], phosphate [19,20,21] and other electrocatalysts have great potential in the field of electrocatalyst application. As an electrocatalyst, the activity of oxygen sulfide is much higher than that of pure oxide or sulfide [22,23,24]. Transition metal selenides and their composite materials have been widely used in catalysis, electrochemistry, and other fields due to their complex physical and chemical properties. Thanks to the metal properties of Se, selenides have higher conductivity than corresponding oxides and sulfides [25,26]. MOOH is generally considered to be an important active intermediate in the oxygen evolution reaction of transition metal selenides [27,28]. Compared with a single metal-based electrocatalyst, a multi-metal-based electrocatalyst shows higher electrocatalytic activity for an oxygen evolution reaction. For example, it has been reported that MoCoNiS is generated by introducing Mo into Co-based electrocatalysts. The polymetallic-based electrocatalysts show excellent oxygen evolution reaction activity and cycle stability, indicating that there is a synergistic effect between different heteroatoms [29]. 

Among transition-metal-based electrocatalysts, Ni-based and Fe-based electrocatalysts have attracted much attention because of their general availability, and the Co element tends to play a role in bimetallic-based electrocatalysts, forming a synergistic effect with other transition metals and bringing about the improvement of catalytic activity for the oxygen evolution reaction [30,31,32]. By introducing other transition metal compounds to form multi-metal-based electrocatalysts, the catalytic activity of the oxygen evolution reaction can be effectively improved. Polymetallic selenides, such as iron-nickel and iron-cobalt selenide, have been proven to be efficient non-noble metal OER electrocatalysts [33,34]. They are in situ transformed into highly active polymetallic oxides in alkaline electrolytes, and synergistic effects occur between different active sites. Du et al. used a one-step hydrothermal method to grow cobalt-selenide-coated nickel selenide nanorods in situ on a nickel foam substrate, which showed excellent OER performance in an alkaline environment. This study confirmed that NiOOH/CoOOH Is instrumental to achieve excellent cycle stability in the alkaline medium in an oxygen evolution reaction, and the synergistic effect between CoSe and NiSe balances the formation of NiOOH/CoOOH heterostructures and exposes more active sites [35]. Liu et al. prepared a Co_X_Ni_1-X_Se_2_ nanoparticle film on a conductive Ti plate using electrodeposition method, which is an excellent bi-functional catalyst. In the selenide formed by Co ions and Ni ions, Co atoms and Ni atoms form a synergistic effect, which significantly improves the oxygen evolution reaction efficiency [36]. Du et al. prepared Fe-doped Ni_3_Se_4_ layered nanosheets using the solvothermal method and topological transformation. During the oxygen evolution reaction, Fe atoms maintained electronic conductivity between the electrode and the metal hydroxide layer, which provides rapid charge transfer and improves the OER activity, with an overpotential of only 225 mV at a current density of 10 mA cm^−2^ [37]. In addition, it was reported that the bond energy of the Co-O bond in OER intermediate CoOOH was reduced by loading Fe_3_O_4_ on the surface of Co_9_S_8_, and the OER activity was effectively increased [38]. Due to the poor conductivity of some transition metal compounds, their development in the field of the electrocatalysis of oxygen evolution reactions is limited. It was reported that the material’s electrical conductivity was improved by combining carbon quantum dots with transition metal compounds, and the reaction rate of oxygen evolution was significantly improved [39]. Hybrid electrocatalysts with highly conductive interconnected carbon frameworks can maintain electrical connections with catalytic active species. Polymetallic selenide carbon matrix composites are expected to improve the electrocatalytic performance.

Based on these studies, a simple one-step hydrothermal method was used to prepare the carbon-quantum-dots-doped polymetallic compound composite with multilayer nanocrystalline sheets (Co_9_Se_8_/Ni_3_Se_4_/Fe_3_O_4_@C). It was found that by constructing heterojunction structures, interface modification can be used to improve the charge transfer rate of materials. The introduction of Fe^2+^ ions can improve the oxygen evolution reaction activity by reducing the bond energy between the reaction active center and the product. At the same time, it was found that the combination of carbon quantum dots and materials brings abundant active sites and conductivity, which leads to higher reactivity. An overpotential of 268 mV at a current density of 10 mA cm^−2^ and a Tafel slope of 64 mV dec^−1^ was shown. These results provide new ideas and suggestions for transition metal selenides to become excellent OER electrocatalysts.

## 2. Experimental Section

### 2.1. Preparation of N-Doped Carbon Quantum Dots Solution

Dissolve 1.05 g citric acid monohydrate and 0.9 g urea in 25 mL distilled water, stir for 30 min, transfer to 100 mL Teflon lined stainless steel autoclave, and keep it warm at 150 °C for 6 h. After cooling to room temperature and centrifuging at 8000 rpm for 10 min, we took the solid part and evenly dispersed it in distilled water to obtain the carbon dots solution.

### 2.2. Preparation of Co_9_Se_8_/Ni_3_Se_4_/Fe_3_O_4_@C

Dissolve 4 g of sodium hydroxide in the mixed solution (10 mL of distilled water and 30 mL of ethanol), add 0.16 g of selenium powder and stir, and heat the mixed solution during stirring until the selenium powder is completely dissolved to form solution A. Dissolve 1.2 mmol cobalt chloride hexahydrate, 0.4 mmol nickel chloride hexahydrate, and 0.4 mmol ferrous sulfate heptahydrate in 0.25 M EDTA solution (solvent is 5 mL distilled water and 5 mL carbon quantum dots solution) to obtain solution B. After mixing solution A and solution B, add 7 mL hydrazine hydrate drop by drop, transfer the mixed solution to 100 mL Teflon lined stainless steel autoclave and keep it warm at 180 °C for 20 h. The black precipitate collected after cooling to room temperature was thoroughly washed with dilute acid and distilled water, then dried at 60 °C for 12 h to obtain the target product.

The samples involved in Co_9_Se_8_/Ni_3_Se_4_/Fe_3_O_4_@C with different Co/Ni/Fe ratios were all prepared using the same experimental method, the metal feeding ratio of the sample (Ni_3_Se_4_/Co_9_Se_8_@C) was Ni:Co = 1:3, and the total amount of metal elements was 2 mmol.

### 2.3. Characterization

The crystal structure of the as-prepared samples was characterized with X-ray diffraction (Rigaku Ultima IV X-ray diffractometer equipped with Cu Ka radiation) at room temperature. The morphology and elemental mapping of the samples were observed through field emission scanning electron microscopy (FESEM, JEOLJSM7800F), with energy dispersive spectrometer (EDS). The crystal structure of the materials was characterized via high-resolution transmission electron microscopy (TEM, JEOLJEM-2100). X-ray photoelectron characterization was recorded on a Thermo scalable 250Xi electron spectrometer system (XPS) using Al Ka radiation. The specific surface area of the sample was measured using Brunauer–Emmett–Teller (BET).

### 2.4. Electrochemical Measurement

All the electrochemical measurements, including LSV, EIS, Cdl, and CP, were carried out in the electrochemical workstation (CHI660E, CH Instrument), using Pt as the counter electrode, Hg/HgO electrode as reference electrode, glass carbon electrode as working electrode, and 1.0 M KOH solution as electrolyte. The working electrode was prepared by adding the 5 mg sample to a mixed solution of 600 μL ethanol, 380 μL ultra-pure water, and 20 μL 5 w% Nafion solution. Then, the mixed solution containing the catalyst sample was ultrasonicated for 30 min to form a well-dispersed suspension. The dispersed catalyst was then dripped onto the glassy carbon electrode (3 mm diameter and 0.35 mg cm^−2^ in loading). According to the Nernst equation (E (RHE) = E (Hg/HgO) + 0.924), the working potential was converted into reversible hydrogen potential, and the OER overpotential was calculated with η (V) = E (RHE) − 1.23 V. For the stability test, 400 μL solution was dropped on the 1 cm × 1 cm foam nickel matrix, dried, and placed on the electrode clamp as the working electrode. Among them, the scanning rate in the linear voltammetry scanning method was 5 mV s^−1^, and the voltage range was 0–0.8 V. The electrochemical active area of samples was evaluated using cyclic voltammetry. The scanning voltage range was 0.1 V, and the scanning speeds were 20 mV s^−1^, 40 mV s^−1^, 60 mV s^−1^, 80 mV s^−1^, and 100 mv s^−1^, respectively. The test voltage for charge transfer resistance was the voltage corresponding to the 10 mA cm^−2^ current density in the polarization curve of the sample, with a frequency of 0.01–100,000 Hz. The test voltage in the stability test was the voltage corresponding to the 10 mA cm^−2^ current density in the polarization curve of the sample.

## 3. Results and Discussion

**Figure 1:**
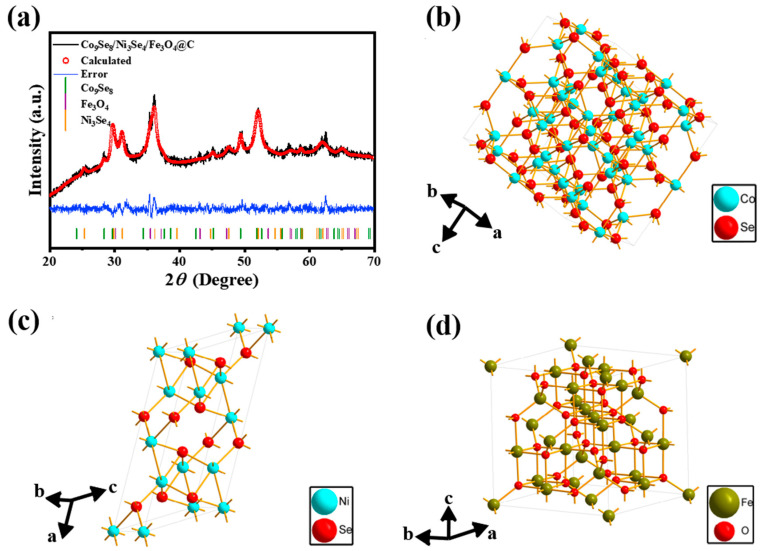
(**a**) XRD patterns of Co_9_Se_8_/Ni_3_Se_4_/Fe_3_O_4_@C; (**b**) the unit cell of Co_9_Se_8_; (**c**) the unit cell of Ni_3_Se_4_; (**d**) the unit cell of Fe_3_O_4_.

Co_9_Se_8_/Ni_3_Se_4_/Fe_3_O_4_@C was synthesized by a simple one-step hydrothermal method (Figure 1a). The Co_9_Se_8_ sample at the XRD diffraction peaks near 28.22°, 29.66°, 45.14°, 49.38°, and 58.56° corresponded to (311), (222), (511), (440) and (622) crystal planes, and the results matched the XRD standard card PDF#09-0233 of Co_9_Se_8_. The Ni_3_Se_4_ component in the sample was the XRD diffraction peaks near 25.28°, 29.76°, 31.14°, 36.19°, 47.70°, 52.07°, 61.89°, and 64.98°, corresponding to (220), (311), (222), (400), (511), (440), (622), and (444) crystal planes; the results matched the XRD standard card PDF#18-0889 of Ni_3_Se_4_. Fe^2+^ ions were oxidized to Fe^3+^ ions by the oxygen in the air. Because the solution was alkaline during the preparation process when the hydrothermal temperature reached 160 °C, Fe ions participated in the reaction, and OH^-^ ions in the solution formed Fe_3_O_4_. The Fe_3_O_4_ component in the sample was at the XRD diffraction peaks near 30.06°, 35.45°, 37.12°, 43.04°, 57.17°, and 62.73°, corresponding to (220), (311), (222), (400), (511), and (440) crystal planes; the results matched the XRD standard card PDF#01-1111 of Fe_3_O_4_. Figure 1b–d show the cell structures of Co_9_Se_8_, Ni_3_Se_4_, and Fe_3_O_4_, in which unsaturated metal atoms involved in surface coordination were exposed to the crystal periphery, providing adsorption active sites for intermediates in electrocatalytic processes [40]. In Figure 1a, the overall peak of XRD shifted to the right because the lattice was doped with larger heteroatoms than the host atoms, which will lead to smaller cell parameters and shift the peak position to the right [41]. Figure S1 shows the XRD diagram of the synthetic samples under different transition metal salt feed ratios. By comparing XRD standard cards, it was found that there was no difference in the chemical composition of the final product synthesized under different metal molar ratios. However, by comparing the corresponding XRD peak intensities of different substances, it was found that the proportion of different chemical components was different.

**Figure 2:**
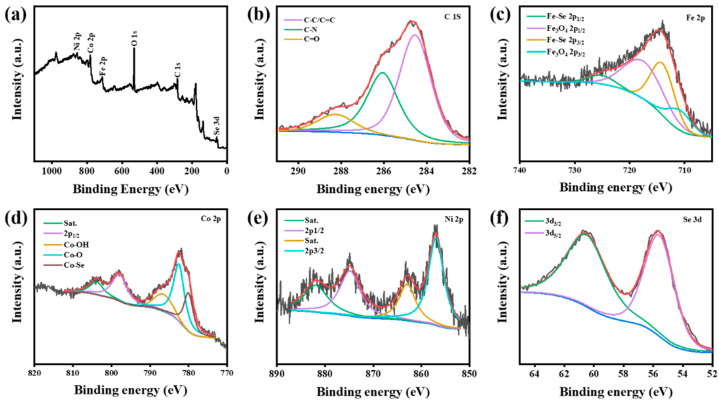
(**a**) XPS spectrum of Co_9_Se_8_/Ni_3_Se_4_/Fe_3_O_4_@C; high-resolution XPS of (**b**) C 1s, (**c**) Fe 2p, (**d**) Co 2p, (**e**) Ni 2p, and (**f**) Se 3d.

In addition, the Co_9_Se_8_/Ni_3_Se_4_/Fe_3_O_4_@C sample was also measured with the XPS measurement to show its near-surface element composition and chemical state. Figure 2b–f show the survey spectrum of the Co_9_Se_8_/Ni_3_Se_4_/Fe_3_O_4_@C sample. According to the attribution of the peaks in the measured spectrum, the chemical compositions of the Co_9_Se_8_/Ni_3_Se_4_/Fe_3_O_4_@C sample in the near-surface range were Ni, Co, Fe, C, and Se (the O element comes from the inevitable air surface adsorption). In multi-component materials, the electronegativity and oxidation states of different elements will cause different changes in the chemical shift and binding energy. The electronegativity of different gold elements is as follows: Fe^3+^ > Ni^2+^ > Co^2+^ > Fe^2+^. The increase in electronegativity will reduce the negative charge density around the atom, which will reduce the shielding effect on the outermost electron, thus leading to the higher binding energy of the atom on the other side of the chemical bond. This will cause the Ni 2p and Co 2p orbitals to shift in the direction of the higher binding energy. At the same time, due to the effect of oxidation, the binding energy of Fe 2p orbitals will also shift in a higher direction.

The results are shown in Figure 2, The signal peak at 803.83 eV represents a satellite peak of Co 2p_1/2_, while the signal peak at 797.92 eV represents a Co 2p_1/2_ orbit. The signal peaks at 786.5 eV and 782.51 eV represent the presence of Co-OH and Co-O bonds, respectively, while the signal peaks at 780 eV represent the Co-Se bonds of the Co_9_Se_8_ phase in Figure 1a [38]. The Ni 2p high-resolution spectrum shows two notable peaks at 874.81 and 856.94 eV, which are assigned to the Ni 2p_1/2_ and Ni 2p_3/2_ signals of Ni, accompanying two shake-up satellite peaks (marked as “Sat.”). This indicated the presence of Ni^3+^ and Ni^2+^ ions (Figure 2e) [42]. The spectrum of Fe 2p consists of two spin orbit states generated by 2p_3/2_ and 2p_1/2_ signals. The signal peaks of Fe at 717.5 eV and 710 eV correspond to the 2p_3/2_ and 2p_3/2_ orbitals of Fe, respectively, and correspond to the mixed oxidation states of Fe^2+^ and Fe^3+^ in Fe_3_O_4_. The signal peaks at 725 eV and 713.9 eV correspond to Fe-Se bonds in the compound, respectively [38]. The signal peaks located at 60.49 eV and 55.59 eV represent Se 3d_3/2_ and Se 3d_5/2_ orbits, respectively (Figure 2f) [43]. Figure 2b shows results expressed as C-C or C=C at 288.25 eV, expressed as C-N at 286.05 eV, interpreted as N element doped in carbon quantum dots, and expressed as C=O at 284.54 eV through the analysis of the XPS spectrum [44]. This is consistent with the XRD results, and it can be judged as the successful synthesis of Co_9_Se_8_/Ni_3_Se_4_/Fe_3_O_4_@C (Figure 1a).

**Figure 3:**
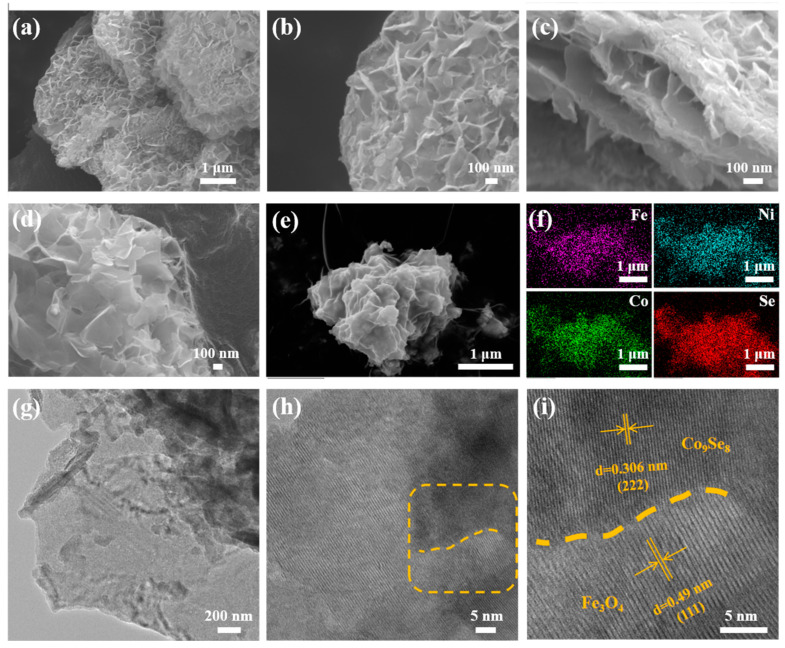
(**a**–**d**) SEM images of Co_9_Se_8_/Ni_3_Se_4_/Fe_3_O_4_@C, (**e**,**f**) EDS elemental mapping of Co_9_Se_8_/Ni_3_Se_4_/Fe_3_O_4_@C, (**g**–**i**) TEM images of Co_9_Se_8_/Ni_3_Se_4_/Fe_3_O_4_@C.

As shown in Figure 3a–d, the prepared catalyst Co_9_Se_8_/Ni_3_Se_4_/Fe_3_O_4_@C presented a multilayer nanosheets layered structure in the micro-size, and had bulges and folds on the surface of the layered structure. Figure 3c shows the layered structure of multilayer nanosheets from another perspective, and the material had a rich pore structure. This provided rich active sites for the catalytic process and effectively improved the catalytic efficiency. As shown in Figure 3e,f, Fe, Co, Ni, and Se were uniformly distributed in the thin sheet, indicating that Co_9_Se_8_, Ni_3_Se_4_, and Fe_3_O_4_ formed uniform thin sheets rather than separating to form their own structure. Because Fe_3_O_4_ has excellent dielectric, magnetic and electrical conductivity, as shown in Figure 3f, the Fe element was uniformly distributed on the material surface, which brought excellent electron transfer efficiency to the catalyst [38].

Figure 3g shows the TEM image of the main sample, C_9_Se_8_, at low magnification, and it can be seen that the sample presents an obvious single-layer nanosheets structure, which is consistent with the multilayer nanosheets structure shown in Figure 3a–d. Under the action of ultrasound, the multilayer nanosheets structure was destroyed and the single-layer nanosheet structure was retained, which shows the size and thickness uniformity of the nanosheets more clearly. Figure 3h,i show the heterojunction structure and local enlargement at high magnification. The lattice stripe spacing of the (222) plane of Co_9_Se_8_ was 0.306 nm, and the lattice stripe spacing of the (111) plane of Fe_3_O_4_ was 0.49 nm. The obvious phase boundary between crystals confirmed the successful formation of the heterojunction. At the same time, it can be seen from Figure 3a–d that there were folds on the surface of the thin section, which also led to an increase in the distance between layers of the multilayer structure, which played a positive role in increasing the specific surface area of the sample. The specific surface area and pore structure of the prepared Co_9_Se_8_/Ni_3_Se_4_/Fe_3_O_4_@C were characterized by nitrogen adsorption/desorption isotherms. Isotherms show porous characteristics (Figure S2). A large specific surface area and porous structure may provide more exposed active sites and accessible channels for electrocatalysis. The specific surface area and pore structure of Co_9_Se_8_/Ni_3_Se_4_/Fe_3_O_4_@C were characterized by nitrogen adsorption/desorption isotherms. Nitrogen adsorption/desorption isotherms are typical type IV isothermal adsorption/desorption curves. The specific surface area reached 32.158 m^2^/g. As shown in Figure S3, the pore size was mainly distributed at about 30 nm, and the larger specific surface area and porous structure of the material may provide more accessible channels for the electrolyte, and more exposed active sites for the electrocatalysis process. 

**Figure 4:**
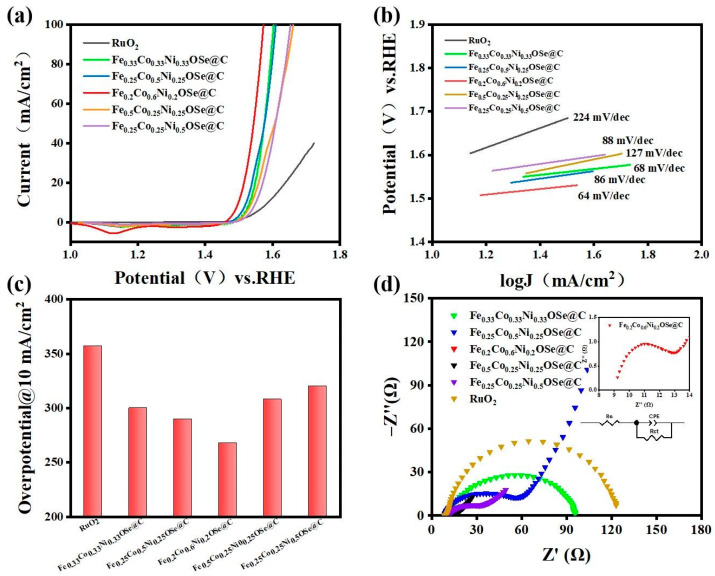
(**a**) The polarization curves of the as-prepared catalyst at a scan rate of 5 mV/s for OER in 1.0 M KOH, (**b**) Tafel plots of as-prepared catalyst in 1.0 M KOH, (**c**) overpotential of different electrocatalysts at 10 mA/cm^2^, (**d**) Nyquist plots of different electrocatalysts.

The electrocatalytic performance of Co_9_Se_8_/Ni_3_Se_4_/Fe_3_O_4_@C was estimated using linear sweep voltammetry (LSV), which uses a three-electrode structure to test in 1.0 M KOH solution. In Figure 4a, redox peaks appear at different potentials. Due to the generation of redox peaks caused by the oxidation of low valence metal ions, Co^2+^ and Ni^3+^ ions changed to a high valence and evolved into Co^3+^ and Ni^4+^ ions. The relationship between the different molar ratios of transition metal FeCoNi and the lowest overpotential was studied, and as shown in Figure 4a, Co_9_Se_8_/Ni_3_Se_4_/Fe_3_O_4_@C (metal element ratio Fe:Co:Ni = 1:3:1) could be found. It exhibited a minimum overpotential of 268 mV at 10 mA/cm^2^, which exceeded that of commercial ruthenium oxide and other samples of different proportions. The corresponding Tafel curves of electrocatalysts with different transition metal element ratios are shown in Figure 4b. Compared with other samples in this study, the Tafel slope of Co_9_Se_8_/Ni_3_Se_4_/Fe_3_O_4_@C (metal element ratio is Fe:Co:Ni = 1:3:1) was 64 mV dec^−1^, which was much lower than commercial ruthenium oxide (224 mV dec^−1^) and other samples with different proportions, indicating that the reaction kinetics were faster. This is because Co_9_Se_8_ and Fe_3_O_4_ form an effective interphase interface engineering. The interface formed by Co_9_Se_8_ and Fe_3_O_4_ can be clearly seen; the upper part is the (222) crystal plane of Co_9_Se_8_ phase, and the lower part is the (111) crystal plane of the Fe_3_O_4_ phase. The obvious boundary of the two phases can be clearly observed in the middle. Thanks to the interface modification, the electron-coupling interaction enhanced the oxygen evolution reaction activity. The oxygen evolution reaction activity of the material has been greatly improved [45,46]. At the same time, the 3D nanosheets ensured a rich exposure of active sites and accelerated the reaction kinetics. The double-layer capacitance was measured using cyclic voltammetry (CV) to estimate the electrochemical surface area (ECSA). A sample with a metal element ratio of Fe:Co:Ni = 1:3:1 had a capacitance of 3.7 mF/cm^2^ (Figures S4–S6). This can be attributed to the participation of Fe and Ni transition metals, as well as the unique structure of the multilayer nanosheets, which brings a larger specific surface area, and the increase in crystal strain caused by the tiny atomic radius difference between nickel and cobalt. The charge transfer resistance (Rct) reflects the difficulty of the step of charge transfer through the two-phase interface between the electrode and electrolyte solution during the electrode process. The electron transfer resistance (Rct) was measured using electrochemical impedance spectroscopy. The Nyquist diagram shows that among the samples with different metal ratios and ruthenium oxide, the sample with the metal element ratio of Fe:Co:Ni = 1:3:1 had the lowest ohmic resistance of 1.44 Ω cm^2^ (Figure 4d). Low Rct means that the electron transfer at the catalyst and electrolyte interface is much faster, and small Rct means good electrical conductivity, both of which are attributed to the combination of carbon quantum dots that improves the overall electrical conductivity of the material.

**Figure 5:**
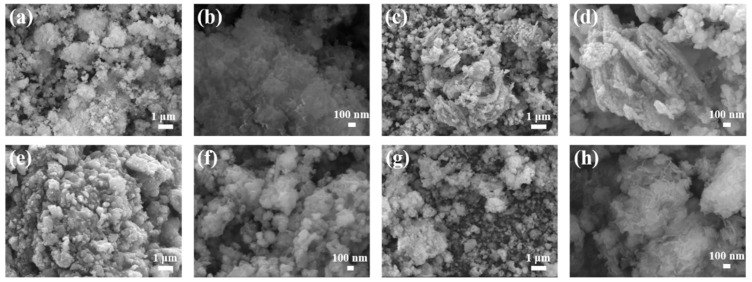
SEM images of Co_9_Se_8_/Ni_3_Se_4_/Fe_3_O_4_@C with different Co/Ni/Fe ratios: (**a**,**b**) Fe_0.33_Co_0.33_Ni_0.33_OSe@C, (**c**,**d**) Fe_0.25_Co_0.5_Ni_0.25_OSe@C, (**e**,**f**) Fe_0.5_Co_0.25_Ni_0.25_OSe@C, (**g**,**h**) Fe_0.25_Co_0.25_Ni_0.5_OSe@C.

The Co_9_Se_8_/Ni_3_Se_4_/Fe_3_O_4_@C electrocatalytic performance was estimated using linear sweep voltammetry (LSV), which was tested in a 1.0 M KOH solution with a three-electrode structure. The relationship between different molar ratios of transition metal FeCoNi and the lowest overpotential was studied. In order to facilitate discrimination, the samples prepared with different metal element ratios were recorded as Fe_X_Co_Y_Ni_Z_OSe@C (for example, a sample prepared with a metal element ratio of Fe:Co:Ni = 1:3:1 was recorded as Fe_0.2_Co_0.6_Ni_0.2_OSe@C). Under the same experimental conditions, with the increase in the Co atoms’ feeding ratio, the minimum overpotential of the sample at 10 mA/cm^2^ current density in LSV gradually decreased and reaches the minimum overpotential when Fe: Co: Ni = 1:3:1, which shows that in Co_9_Se_8_/Ni_3_Se_4_/Fe_3_O_4_@C for the oxygen evolution reaction on the sample, its catalytic active center should be the position of Co atoms, but the excessive ratio of Co atoms weakened the synergistic effect between Fe, Co and Ni, so there was an optimal ratio (Figure 4a). With the increase in the proportion of Co atoms, the micromorphology of the samples evolved from nanoparticles to multilayer nanoflakes (Figure 5a–d). In order to display the microstructure of the sample more clearly, an enlarged image has been added in Figure S7 to support the information. At the optimal ratio of Fe:Co:Ni = 1:3:1, the morphology of the sample presented a structure of multilayer nanosheets, which provided a better scheme for full contact with the electrolyte.

When Fe or Ni elements are dominant metal elements, OER performance is found to be reduced, which further confirms that the Co group is the active site for the oxygen evolution reaction of samples. This is preliminarily consistent with the oxygen evolution reaction pathway of the cobalt oxygen catalyst proposed by Mattioli et al. In several intermediate steps of the oxygen evolution reaction, the last O_2_ molecule is separated from the Co active center, which may be the speed control step of cobalt-based catalysts [35]. The dominant position of the Fe atoms will lead to the synthesis of more particle structures in the micromorphology, which is not conducive to the increase in specific surface area and the exposure of the active site. The dominant position of Ni atoms will lead to the formation of flower-like nanosheets, which is also not conducive to the full contact between the active site and the electrolyte (Figure 5e–h). Studies have shown that the OER performance of Co_9_S_8_ grown on the go was significantly improved after the introduction of Fe on the surface of Co_9_S_8_ [38]. Se atoms and S atoms have the same outermost electron distribution and a similar electronic structure. Fe_3_O_4_ has a mixed oxidation state of Fe^3+^ and Fe^2+^ ions, in which Fe^2+^ ion has an electronegativity of 1.83, lower than the 1.88 of Co^2+^ ions. Electronegativity reflects the ability of atoms in elements to attract electrons in compounds. During the polarization process, Co^2+^ ions attract electrons around Fe^2+^, causing Co to be in a low oxidation state, and thereby promoting the fracture of Co-O bonds and the release of O_2_, accelerating the activity of oxygen evolution reactions [32]. This also confirmed that with the participation of the Fe element, the OER performance of samples was improved, but the increase in the Fe component would lead to the reduction in Co species as catalytic active centers, and the reduction of exposed active sites would lead to the decline in OER performance. It is well-known that Fe^3+^ is the strongest transition metal based on Lewis acid, and that Fe^3+^ ions have remarkable effects on changing the electronic properties of other metal cations. Ni species will be oxidized after polarization to form more Ni^4+^ cations, which will reduce the catalytic efficiency of the oxygen evolution reaction. The participation of Fe^3+^ ions will effectively inhibit the dynamic competition of the metal oxidation effect, which will lead to the enhancement of oxygen evolution reaction activity [47]. Ni atoms and Co atoms are involved in the formation of selenides, and the presence of Ni atoms makes the lattice of Co_9_Se_8_ slightly offset, which reduces the activation energy of intermediates generated in the OER process and improves the catalytic efficiency.

**Figure 6:**
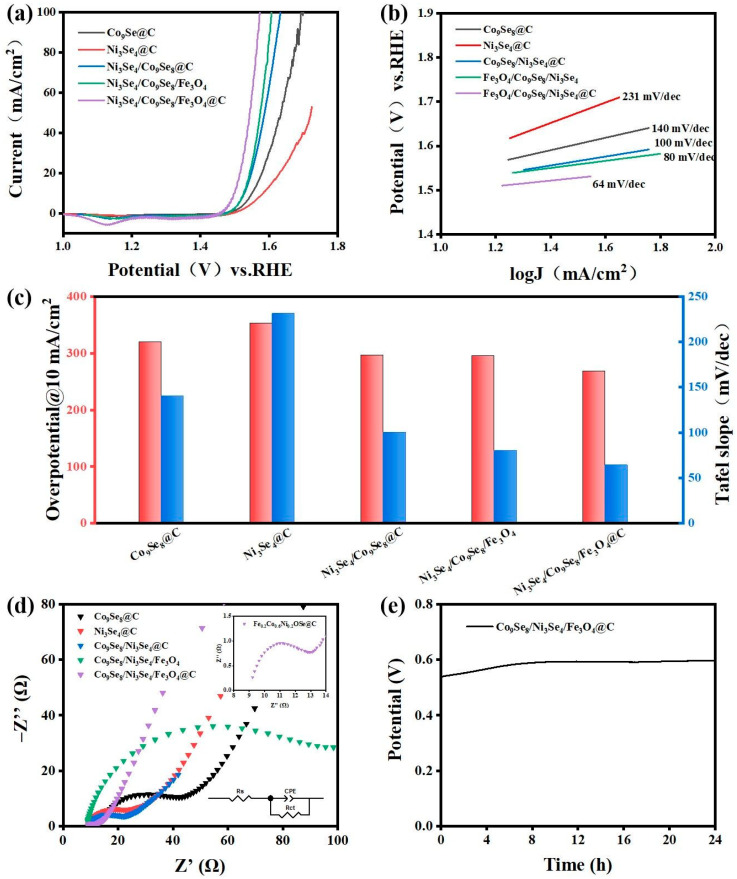
(**a**) The polarization curves of the as-prepared catalyst at a scan rate of 5 mV/s for OER in 1.0 M KOH, (**b**) Tafel plots of the as-prepared catalyst in 1.0 M KOH, (**c**) The overpotential and Tafel slope of the prepared catalyst in 1.0 M KOH at a scanning rate of 10 mV/s, (**d**) Nyquist plots of different electrocatalysts, (**e**) the voltage-time curve of the sample in 1.0 M KOH solution at a constant current density of 10 mA/cm^2^ for 24 h.

In order to analyze the role of different components in composite materials, single/multi-component samples were successfully synthesized using the same experimental method for comparison (Figure S8). The oxygen evolution reaction parameters of the material were tested with a simple three-electrode system in a 1.0 M KOH solution (Figure 6a–c). The catalytic activity of single metal and bimetallic electrocatalysts was significantly worse than that of a multi-metal-based electrocatalyst. This was attributed to the interface modification, which enhanced the activity of the oxygen evolution reaction due to the electron-coupling interaction. In Figure 6a, the introduction of Fe_3_O_4_ brings an obvious boost to the catalytic activity of the catalyst for the oxygen evolution reaction, which is attributed to the enhanced electron transport efficiency by Fe_3_O_4_, as well as the reduced bond energy of the oxygen evolution reaction intermediate MOOH. The participation of Fe_3_O_4_ can effectively inhibit the dynamic competition of the metal oxidation effect; that is, via the transition of Ni ions to high valence during the polarization process. This will increase the catalytic activity of the oxygen evolution reaction. At the same time, carbon quantum dots have a large number of active site due to their rich heteroatoms. The combination with the catalyst material brings about active sites for oxygen evolution reaction, which better improves the overall electron transport efficiency of the material [10]. The introduction of carbon quantum dots can significantly reduce the resistivity of the material, and the combined effect of Fe_3_O_4_ and carbon quantum dots effectively improves the overall electron transfer efficiency of the material (Figure 6d). Figure 6c shows the overpotential and Tafel slope histogram of different materials at a current density of 10mA/cm^2^, which can more intuitively display the oxygen evolution reaction activity and reaction kinetics of different materials. The electric double-layer capacitance of different samples was measured and calculated using cyclic voltammetry (Figures S9–S12). It was found that the introduction of carbon quantum dots will reduce the electric double-layer capacitance of the product, but it will have a better catalytic effect on the oxygen evolution reaction. In order to evaluate the chemical stability of the electrocatalyst, the material was kept in a 1.0 M KOH solution for 24 h at a constant current density of 10 mA/cm^2^, and the material showed excellent stability (Figure 6e).

After 24 h of the stability test, the micro-morphology of the sample changed significantly (Figure S13). The multilayer nanosheets structure collapsed during the long time of polarization, and the thickness of the lamellar structure increased, which led to less contact with the electrolyte. This was the main reason for the increase in the polarization voltage during the stability test. After the XPS test of the sample after the stability test (Figure S14), it was found that Se evolves into SeO_x_ species under strong polarization, and the signal peak of SeO_x_ can be observed, which was caused by the oxidation of Se under alkaline conditions. The signal peak of the Fe element had no obvious change before and after the test, which was due to the good stability of Fe^3+^ ions in the oxygen evolution reaction process, as the strongest Lewis acid [47]. In addition, the electrons of Ni and Co atoms could be transferred to Fe atoms to protect them from oxidation and transition to a high oxidation state [32].

## 4. Conclusions

In summary, the Co_9_Se_8_/Ni_3_Se_4_/Fe_3_O_4_@C with a multilayer nanosheets structure prepared with a simple one-step hydrothermal method had an overpotential of 268 mV and a Tafel slope of 64 mV dec^−1^ at a current density of 10 mA cm^−2^. First, the polymetallic selenide formed a highly active polymetallic oxide in the alkaline electrolyte, resulting in a synergistic effect of different active sites. Then, due to the structure of the multilayer nanosheets, more active sites were generated. The dots were exposed, which greatly improved the catalytic efficiency. Through the formation of effective interface engineering between the two phases, the oxygen evolution reaction activity of the material was greatly improved. Due to the interface modification, the electron-coupling interaction enhanced the oxygen evolution reaction activity, and the 3D nanosheets ensured a rich exposure of active sites and accelerated the reaction kinetics. Finally, since carbon quantum dots have abundant heteroatoms, they can provide more active sites for the oxygen evolution reaction. After being compounded with transition metal selenides, they also improved the conductivity of the materials and the electron transfer efficiency in the catalytic process. This provides new insights into design ideas for the future synthesis of high-efficiency and low-cost electrocatalysts.

## Data Availability

No new data were created or analyzed in this study. Data sharing is not applicable to this article.

## Supplementary Materials

The following supporting information can be downloaded at: https://www.mdpi.com/article/10.3390/ma16145115/s1, Figure S1: XRD map of Co_9_Se_8_/Ni_3_Se_4_/Fe_3_O_4_@C with different Co/Ni/Fe ratios; Figure S2: Nitrogen constant temperature adsorption-desorption curve of Co_9_Se_8_/Ni_3_Se_4_/Fe_3_O_4_@C; Figure S3: Pore size distribution of Co_9_Se_8_/Ni_3_Se_4_/Fe_3_O_4_@C; Figure S4: CV curves of Fe_0.33_Co_0.33_Ni_0.33_OSe@C; Figure S5: CV curves of Fe_0.25_Co_0.5_Ni_0.25_OSe@C; Figure S6: CV curves of Fe_0.2_Co_0.6_Ni_0.2_OSe@C; Figure S7: SEM images of Co_9_Se_8_/Ni_3_Se_4_/Fe_3_O_4_@C with different Co/Ni/Fe ratios: (a,b) Fe_0.33_Co_0.33_Ni_0.33_OSe@C, (c,d) Fe_0.25_Co_0.5_Ni_0.25_OSe@C, (e,f) Fe_0.5_Co_0.25_Ni_0.25_OSe@C, (g,h) Fe_0.25_Co_0.25_Ni_0.5_OSe@C; Figure S8: XRD patterns of single/multi-component samples prepared using the same experimental method; Figure S9: CV curves of Co_9_Se_8_@C; Figure S10: CV curves of Ni_3_Se_4_@C; Figure S11: CV curves of Co_9_Se_8_/Ni_3_Se_4_@C; Figure S12: CV curves of Fe_3_O_4_/Co_9_Se_8_/Ni_3_Se_4_; Figure S13: SEM images of primary samples after 24 h stability testing; Figure S14: XPS spectrum of main samples after stability test: (a) Co 2p, (b) Fe 2p, (c) Ni 2p, (d) Se 1s; Table S1: The comparison between this work and other similar work in recent years on the activity of oxygen evolution reaction. References [13,48,49,50,51,52,53,54,55,56,57,58,59,60,61] are cited in the supplementary materials.
