# Supplementary material for: Carbon Quantum Dots-Doped Ni3Se4/Co9Se8/Fe3O4 Multilayer Nanosheets Prepared Using the One-Step Solvothermal Method to Boost Electrocatalytic Oxygen Evolution
Source: Materials (Basel). 2023 Jul 20;16(14):5115. doi: 10.3390/ma16145115 (PMC10383042; doi:10.3390/ma16145115)
Supplement: Supplementary file 1 [file materials-16-05115-s001.zip › materials-2434540-supplementary.pdf]

Supporting Information

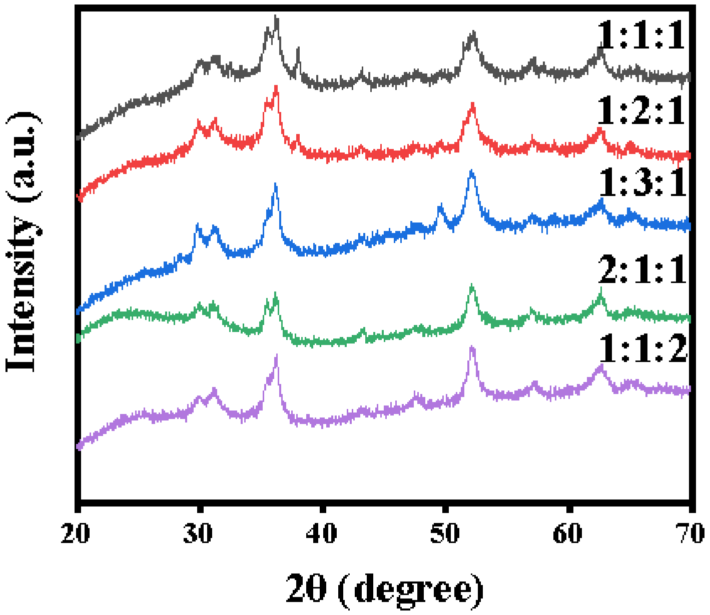

Figure S1. XRD map of $Co_9Se_8/Ni_3Se_4/Fe_3O_4$@C with different Co/Ni/Fe ratios.

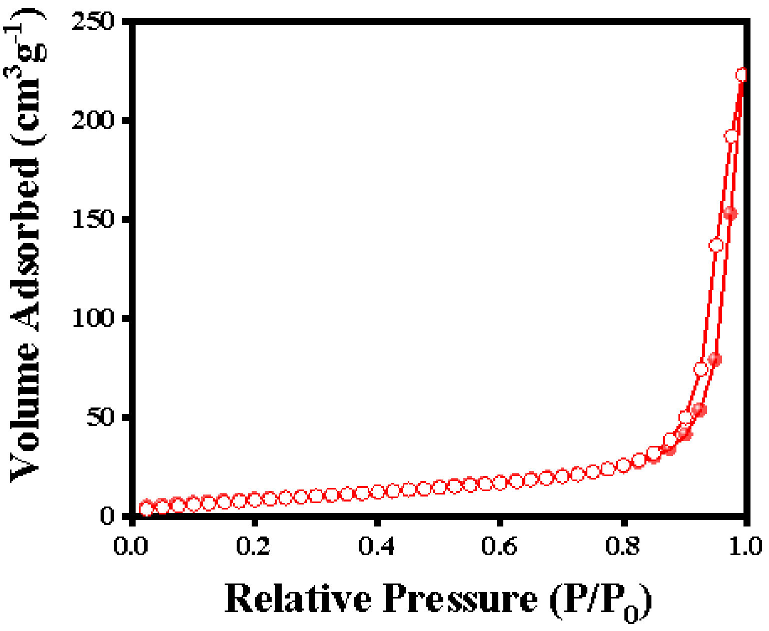

Figure S2. Nitrogen constant temperature adsorption–desorption curve of $Co_9Se_8/Ni_3Se_4/Fe_3O_4$@C.

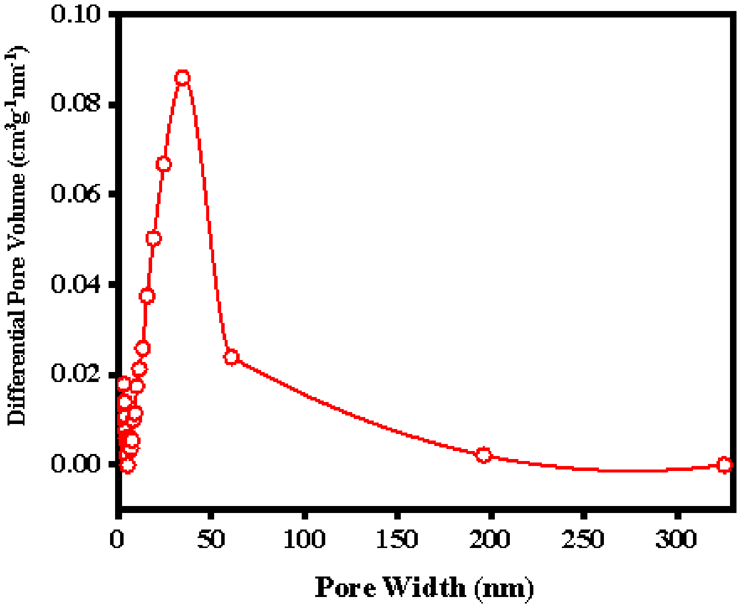

Figure S3. Pore size distribution of $Co_9Se_8/Ni_3Se_4/Fe_3O_4@C$.

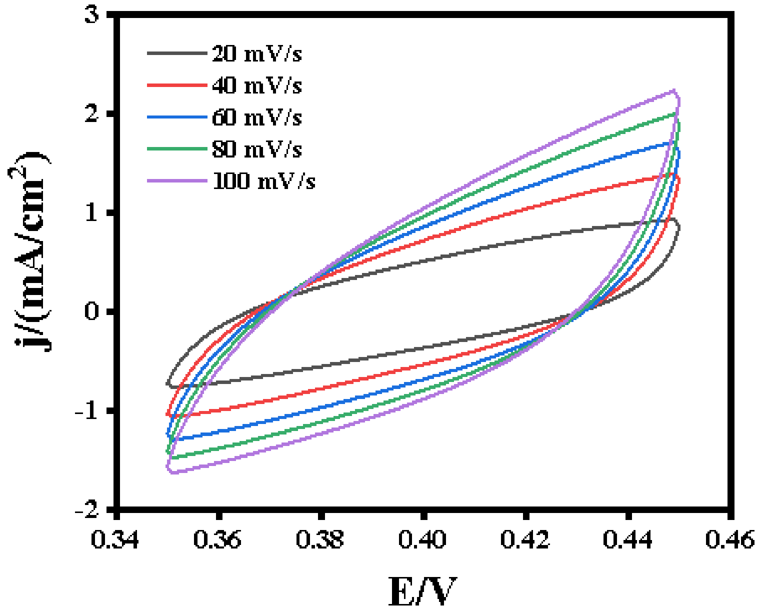

Figure S4. CV curves of $Fe_{0.33}Co_{0.33}Ni_{0.33}OSe@C$.

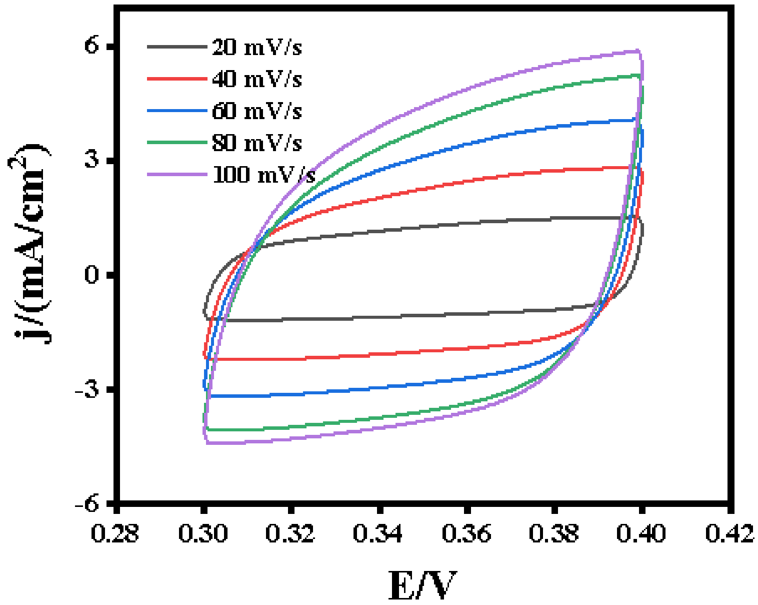

Figure S5. CV curves of $Fe_{0.25}Co_{0.5}Ni_{0.25}OSe@C$.

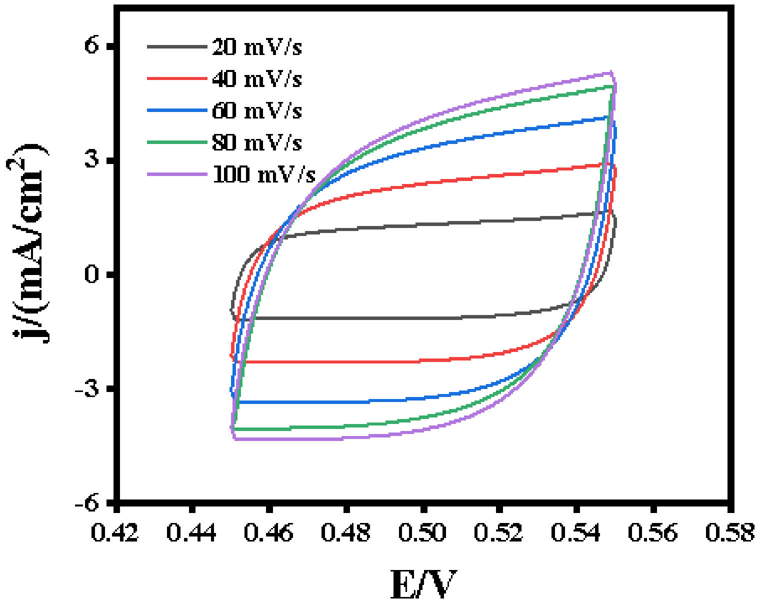

Figure S6. CV curves of $Fe_{0.2}Co_{0.6}Ni_{0.2}OSe@C$.

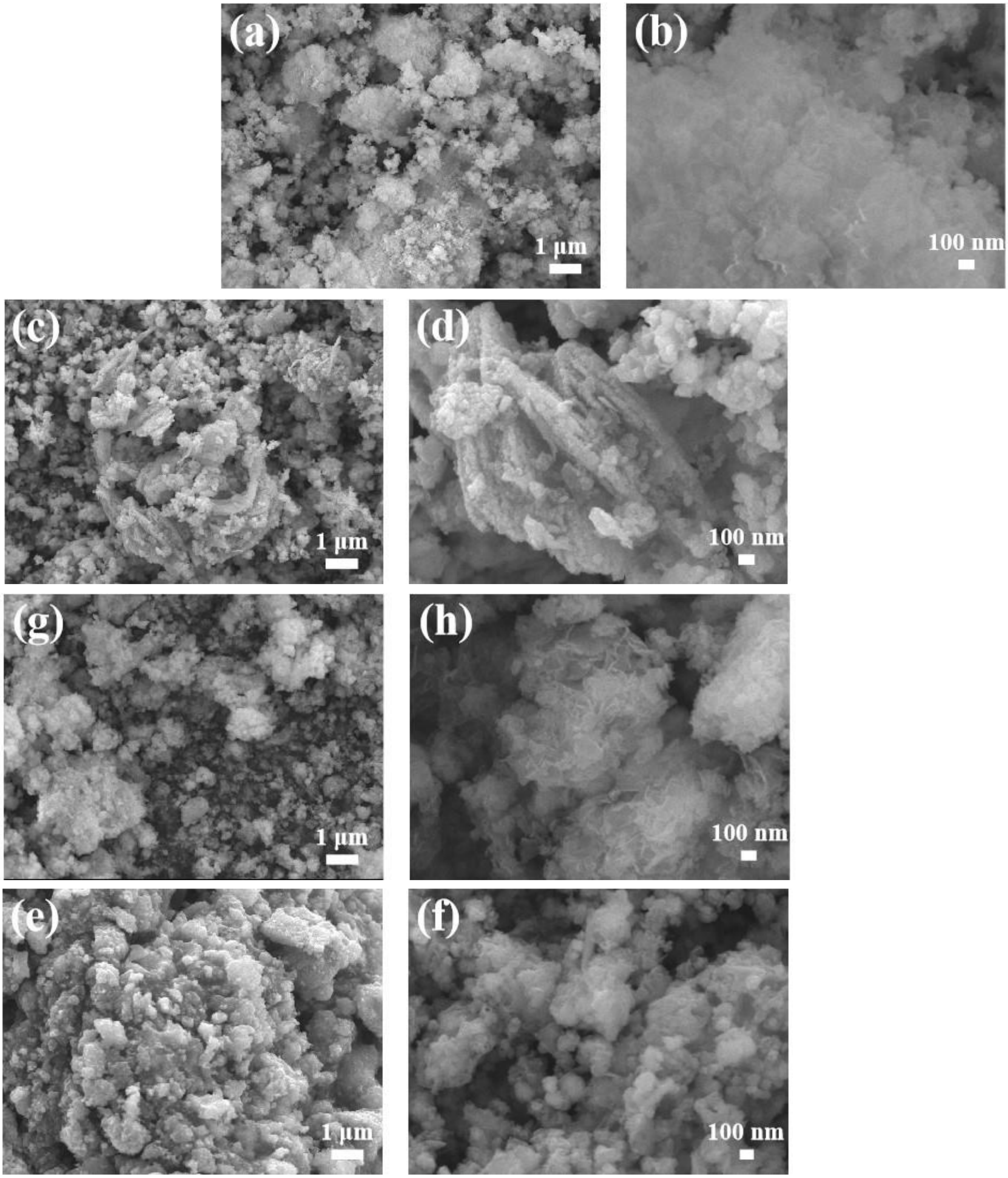

Figure S7. SEM images of $Co_9Se_8/Ni_3Se_4/Fe_3O_4$@C with different Co/Ni/Fe ratios: (a,b) $Fe_{0.33}Co_{0.33}Ni_{0.33}OSe$@C, (c,d) $Fe_{0.25}Co_{0.5}Ni_{0.25}OSe$@C, (e,f) $Fe_{0.5}Co_{0.25}Ni_{0.25}OSe$@C, (g,h) $Fe_{0.25}Co_{0.25}Ni_{0.5}OSe$@C.

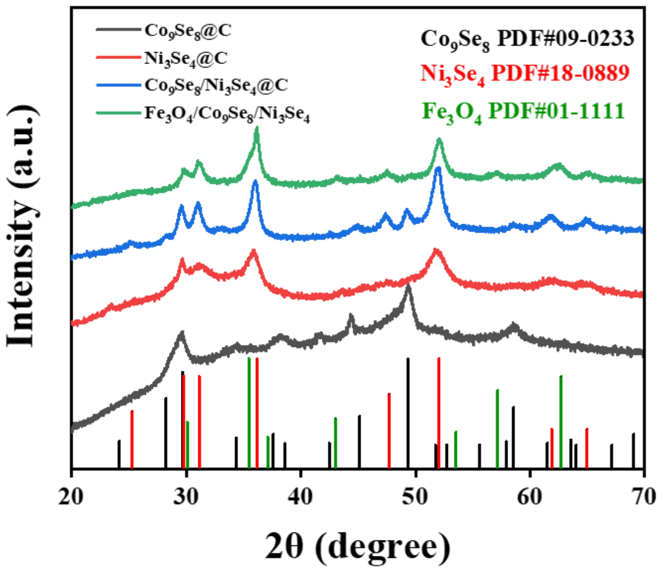

Figure S8. XRD patterns of single/multi-component samples prepared using the same experimental method.

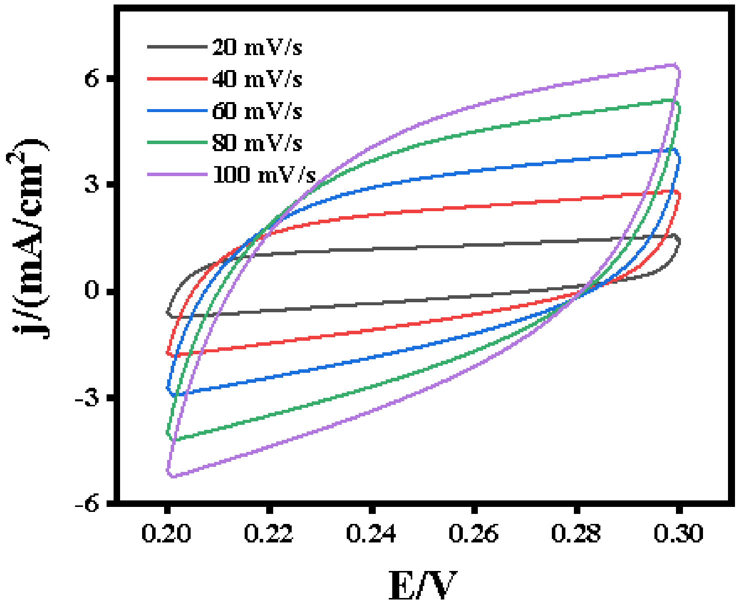

Figure S9. CV curves of Co_9Se_8@C.

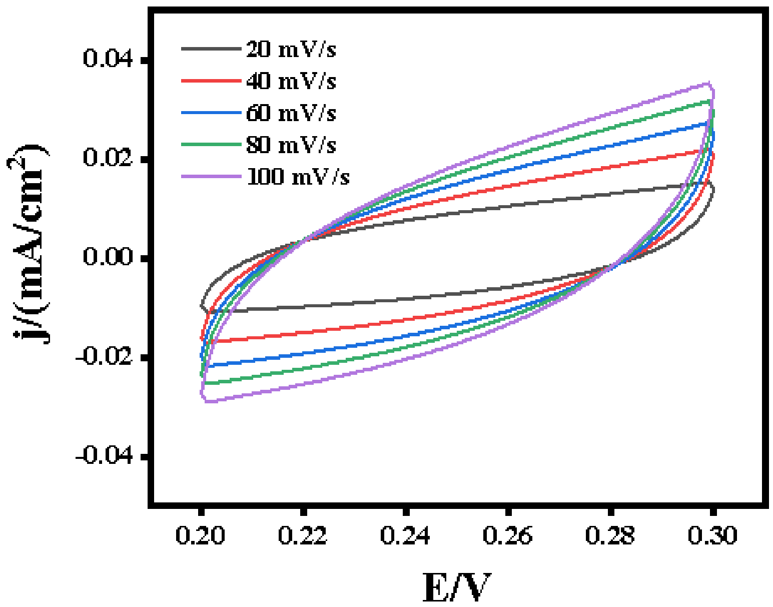

Figure S10. CV curves of Ni_3Se_4@C.

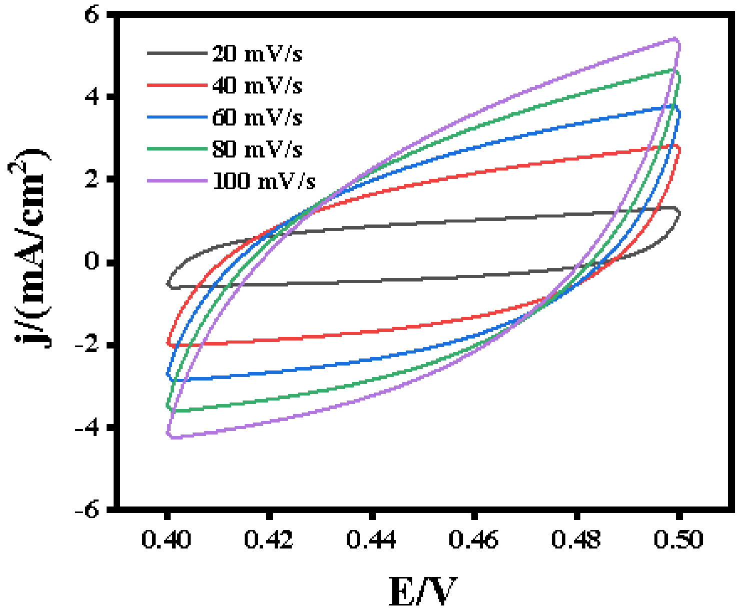

Figure S11. CV curves of Co_9Se_8/Ni_3Se_4@C.

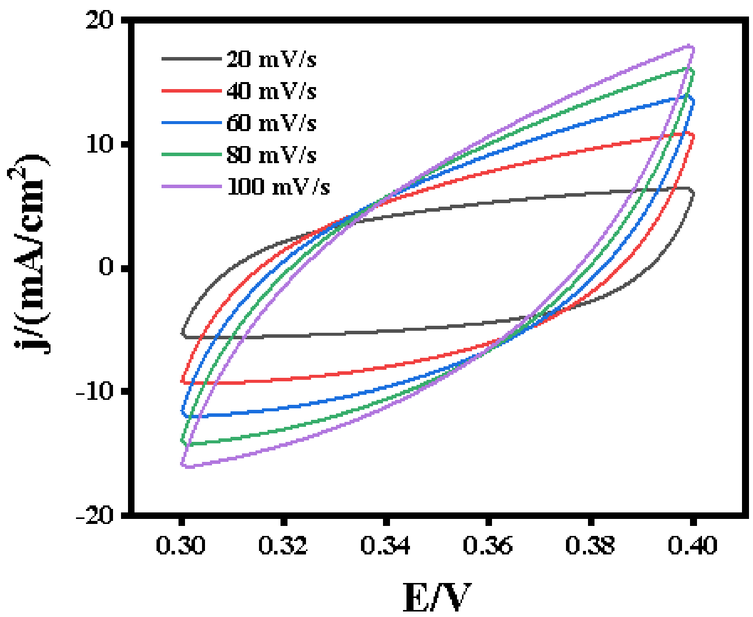

Figure S12. CV curves of $Fe_3O_4/Co_9Se_8/Ni_3Se_4$.

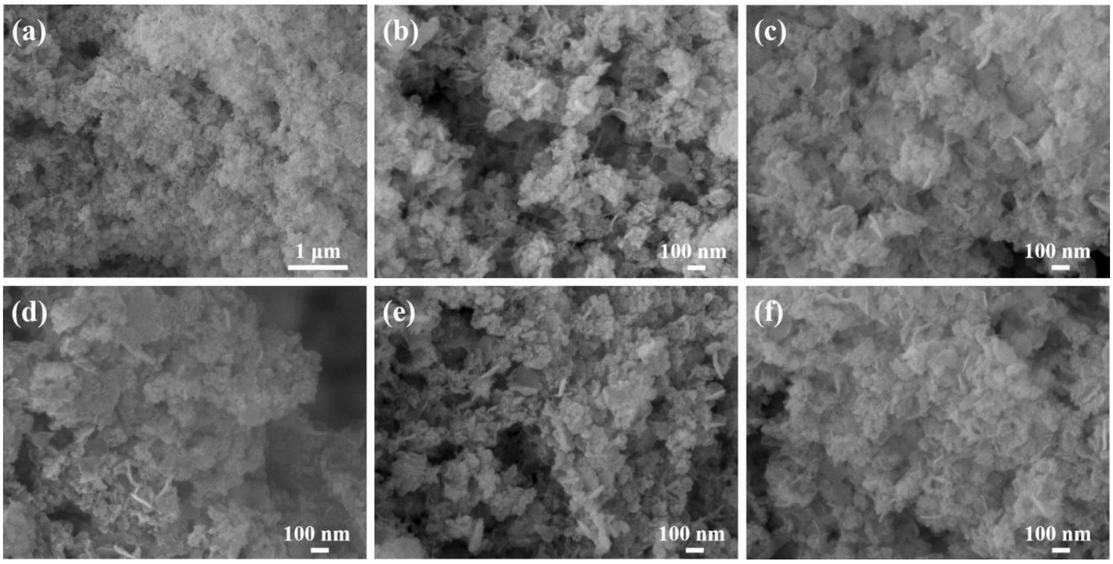

Figure S13. SEM images of primary samples after 24 h stability testing.

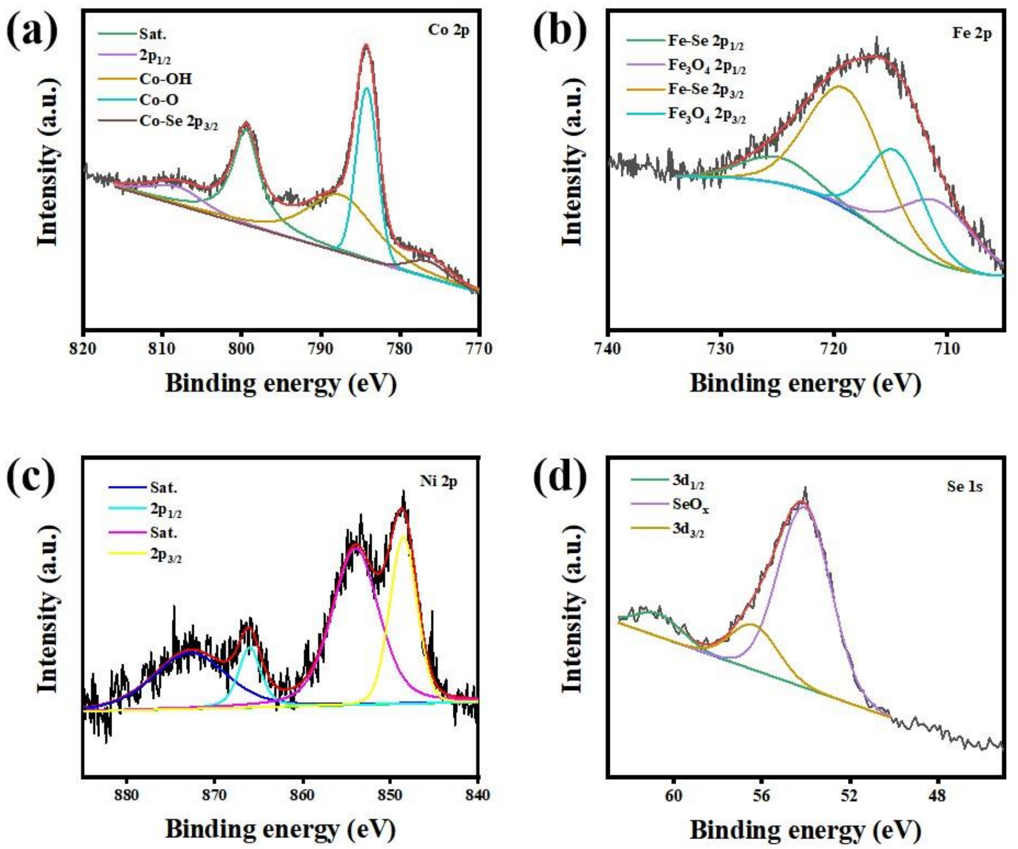

Figure S14. XPS spectra of main samples after stability test: (a) Co 2p, (b) Fe 2p, (c) Ni 2p, (d) Se 1s.

Table S1. The comparison between this work and other similar work in recent years on the activity of oxygen evolution reaction.

Catalyst	η at 10 mA cm^{-2} (mV)	Tafel Slope (mV dec^{-1})	Reference
$Co_9Se_8/Ni_3Se_4/Fe_3O_4$@C	268	64	This work
a-CoSe/Ti	292	69	1
Coral-like CoSe	295	40	2
$Ni_{0.5}Co_{0.5}Se$	216	37	3
WCoSe/WCo_3O_4	175	62	4
$Co_{0.85}Se$-NC/C-750	298	47	5
P-$NiSe_2$@N-CNTs/NC hybrid	306	61	6
$CoSe_2$@Fe-CoO hybrid	280	56	7
(Ni,Fe)Se@NiFe-LDH/NF	253(η_{100})	47	8
$CoSe_2/FeSe_2$@C	290	62	9
Fe-Co_2P@NPDC	320	61	10
high-active Co_3O_4	268	74	11
$NiCo_2S_4$/CoFeMo-LDH	295(η_{500})	83	12
B,N-GQDs/MOF-derived-NiFe-LDH	251(η_{100})	35	13
Fe-doped Ni_3S_2/FeS_2	230(η_{100})	32	14
Fe-CoP@Ni_2P	237(η_{50})	51	15

References

1. Liu, T.T.; Liu, Q.; Asiri, A.M.; Luo, Y.L.; Sun, X.P. An amorphous CoSe film behaves as an active and stable full water-splitting electrocatalyst under strongly alkaline conditions. *Chem. Commun.* **2015**, *51*, 16683–16686. https://doi.org/10.1039/C5CC06892D.
2. Liao, M.; Zeng, G.F.; Luo, T.T.; Jin, Z.Y.; Wang, Y.J.; Kou, X.M.; Xiao, D. Three-dimensional coral-like cobalt selenide as an advanced electrocatalyst for highly efficient oxygen evolution reaction. Electrochim. Acta. **2016**, *194*, 59–66. http://doi.org/10.1016/j.electacta.2016.02.046.
3. Zhu, L.; Liao, Y.X.; Jia, Y.B.; Zhang, X.; Ma, R.G.; Wang, K.K. Solid-solution hexagonal $Ni_{0.5}Co_{0.5}Se$ nanoflakes toward the boosted oxygen evolution reaction. *Chem Commun.* **2020**, *56*, 13113–13116. https://doi.org/10.1039/D0CC05247G.
4. Balaji, R.; Nguyen, T.T.; Harish, K.; Kim, N.H.; Lee, J.H. Modulating heterointerfaces of tungsten incorporated CoSe/Co_3O_4 as a highly efficient electrocatalyst for overall water splitting. *J. Mater. Chem. A* **2022**, *10*, 3782–3792. http://doi.org/10.1039/d1ta09932a.
5. Han, Y.; Chen, X.; Qian, C.; Zhang, X.Y.; He, W.; Ren, H.J.; Li, H.B.; Diao, G.W.; Chen, M. $Co_{0.85}Se$ nanoparticles armored by N-doped carbon layer with electronic structure regulation functions: An efficient oxygen evolution electrocatalyst. *Chem. Eng. J.* **2021**, *420*, 130461. https://doi.org/10.1016/j.cej.2021.130461.
6. Yu, J.; Li, W.J.; Kao, G.B.; Xu, C.Y.; Chen, R.R.; Liu, Q.; Liu, J.Y.; Zhang, H.S.; Wang, J. In-situ growth of CNTs encapsulating P-doped $NiSe_2$ nanoparticles on carbon framework as efficient bifunctional electrocatalyst for overall water splitting. *J. Energy Chem.* **2021**, *60*, 111–120. https://doi.org/10.1016/j.jechem.2020.12.030.
7. Ramadoss, M.; Chen, Y.F.; Hu, Y.; Wang, B.; Jeyagopal, R.; Marimuthu, K.; Wang, X.Q.; Yang, D.X. Hierarchically porous nanoarchitecture constructed by ultrathin $CoSe_2$ embedded Fe-CoO nanosheets as robust electrocatalyst for water oxidation. *J. Mater. Sci. Technol.* **2021**, *78*, 229–237. https://doi.org/10.1016/j.jmst.2020.10.058.
8. Hu, J.; Liang, Y.Q.; Wu, S.L.; Li, Z.Y.; Shi, C.S.; Luo, S.Y.; Sun, H.J.; Zhu, S.L.; Cui, Z.D. Hierarchical nickle-iron layered double hydroxide composite electrocatalyst for efficient oxygen evolution reaction. *Mater. Today Nano.* **2022**, *17*, 100150. https://doi.org/10.1016/j.mtnano.2021.100150.

9. Li, W.; Niu, Y.; Wu, X.; Wu, F.; Li, T.; Hu, W. Heterostructured $CoSe_2$/$FeSe_2$ Nanoparticles with Abundant Vacancies and Strong Electronic Coupling Supported on Carbon Nanorods for Oxygen Evolution Electrocatalysis. *ACS Sustainable Chem. Eng.* **2020**, *11*, 4658–4666. https://doi.org/10.1021/acssuschemeng.0c00839.
10. Li, L.H.; Zhang, L.; Nie, Z.C.; Ma, W.Y.; Li, N.P.; Wågberg, T.; Hu, G.Z. Tailoring charge reconfiguration in dodecahedral Co_2P@carbon nanohybrids by triple-doping engineering for promoted reversible oxygen catalysis. *J. Mater. Chem. A* **2022**. https://doi.org/10.1039/D2TA04482J.
11. Zhang, T.Y.; Zhao, S.C.; Zhu, C.M.; Shi, J.; Su, C.; Yang, J.W.; Wang, M.; Li, J.; Li, J.H.; Liu, P.L.; et al. Rational construction of high-active Co_3O_4 electrocatalysts for oxygen evolution reaction. *Nano Res.* **2023**, *16*, 624–633. https://doi.org/10.1007/s12274-022-4879-2.
12. Shen, X.R.; Li, H.J.; Zhang, Y.Y.; Ma, T.T.; Li, Q.; Jiao, Q.Z.; Zhao, Y.; Li, H.S.; Feng, C.H. Construction dual-regulated $NiCo_2S_4$ @Mo-doped CoFe-LDH for oxygen evolution reaction at large current density. *Appl. Catal. B* **2022**, *319*, 121917. https://doi.org/10.1016/j.apcatb.2022.121917.
13. Rinawati, M.; Wang, Y.X.; Huang, W.H.; Wu, Y.T.; Cheng, Y.S.; Kurniawan, D.; Haw, S.C.; Chiang, W.H.; Su, W.N.; Yeh, M.H. Unraveling the efficiency of heteroatom-doped graphene quantum dots incorporated MOF-derived bimetallic layered double hydroxide towards oxygen evolution reaction. Carbon. **2022**, *200*, 437–447. https://doi.org/10.1016/j.carbon.2022.08.067.
14. Gultom, N.S.; Li, C.H.; Kuo, D.H.; Abdullah, H. Single-Step Synthesis of Fe-Doped Ni_3S_2/FeS_2 Nanocomposites for Highly Efficient Oxygen Evolution Reaction. *ACS Appl. Mater. Interfaces.* **2022**, *14*, 39917–39926. https://doi.org/10.1021/acsami.2c08246.
15. Duan, Z.X.; Zhao, D.P.; Sun, Y.C.; Tan, X.J.; Wu, X. Bifunctional Fe-doped CoP@Ni_2P heteroarchitectures for high-efficient water electrocatalysis. *Nano Res.* **2022**, *15*, 8865–8871. https://doi.org/10.1007/s12274-022-4673-z.
